# The Effects of Air Pollution on COVID-19 Related Mortality in Northern Italy

**DOI:** 10.1007/s10640-020-00486-1

**Published:** 2020-08-04

**Authors:** Eric S. Coker, Laura Cavalli, Enrico Fabrizi, Gianni Guastella, Enrico Lippo, Maria Laura Parisi, Nicola Pontarollo, Massimiliano Rizzati, Alessandro Varacca, Sergio Vergalli

**Affiliations:** 1 College of Public Health and Health Professions, University of Florida, Gainesville, FL USA; 2 Fondazione Eni Enrico Mattei, Milan, Italy; 3 Department of Economics and Social Sciences, Università Cattolica del Sacro Cuore, Piacenza, Italy; 4 Department of Mathematics and Physics, Università Cattolica del Sacro Cuore, Brescia, Italy; 5 Department of Economics and Management, Università degli Studi di Brescia, Brescia, Italy; 6 Department of Agricultural Economics, Università Cattolica del Sacro Cuore, Piacenza, Italy

**Keywords:** COVID-19, Mortality, Pollution, Italy, Municipalities, Q53, I18, J11

## Abstract

Long-term exposure to ambient air pollutant concentrations is known to cause chronic lung inflammation, a condition that may promote increased severity of COVID-19 syndrome caused by the novel coronavirus (SARS-CoV-2). In this paper, we empirically investigate the ecologic association between long-term concentrations of area-level fine particulate matter (PM_2.5_) and excess deaths in the first quarter of 2020 in municipalities of Northern Italy. The study accounts for potentially spatial confounding factors related to urbanization that may have influenced the spreading of SARS-CoV-2 and related COVID-19 mortality. Our epidemiological analysis uses geographical information (e.g., municipalities) and negative binomial regression to assess whether both ambient PM_2.5_ concentration and excess mortality have a similar spatial distribution. Our analysis suggests a positive association of ambient PM_2.5_ concentration on excess mortality in Northern Italy related to the COVID-19 epidemic. Our estimates suggest that a one-unit increase in PM_2.5_ concentration (µg/m^3^) is associated with a 9% (95% confidence interval: 6–12%) increase in COVID-19 related mortality.

## Introduction

With more than twelve million confirmed COVID-19 cases and more than 550 thousand related deaths globally as of the beginning of July 2020,^1^ the novel coronavirus pandemic has unquestionably caused dramatic health and economic impacts. Despite the public health benefits of the consequent COVID-19 mitigation measures adopted by the central and the regional governments in Italy, one of the most heavily impacted countries, there are adverse socioeconomic effects of the lockdown on top of what are already dramatic public health impacts. Official morbidity statistics, although complicated by the public health interventions and the emergency status, reveal a strong spatial clustering phenomenon across administrative regions in Italy and provinces and municipalities within each region. Such a geographical concentration of both COVID-19 morbidity and mortality is most likely the result of the interaction of multiple factors, among which include the clustering of initially infected individuals, different choices made about testing and contact tracing in order to identify community transmission, underlying population demographic and prevalence of health status, and the timely adoption of lockdown measures to control the COVID-19 epidemic (Ciminelli and Garcia-mandicó 2020). Beyond such proximal factors, however, additional contextual factors may have played an important role in the health impacts of COVID-19 in Italy.

The Northern Italian regions most affected by the spreading of coronavirus (Lombardia, Veneto, Piemonte, Emilia Romagna) are also the most densely populated and heavily industrialized and thereby the most heavily polluted regions of Italy. These four regions together host 39% of the national population,^2^ and approximately one-half of the Italian GDP is produced there. Such a spatial concentration of economic activities involves the industrial manufacturing sectors to the largest extent, and the consequent high level of emissions is at least in part responsible for poor air quality in the region.^3^ In Brescia, among the most affected cities in Lombardy, the concentration of particulate matter (PM) and ozone exceeded the allowable threshold in 150 days in 2018, making it the most polluted city in Italy. Lodi and Monza follow, with 149 and 140 exceedance days, respectively. Milan and Bergamo are sixth and ninth, respectively, with 135 and 127 days.^4^ Lombardy is also among the most polluted regions in all of Europe (European Environmental Agency 2019). The relatively higher air pollutant concentrations in the Po Valley region of Italy contrasts sharply with neighbouring alpine regions and stems from the combination of two main factors (Carugno et al. 2016; Larsen et al. 2012; Pozzer et al. 2019). The first is the high concentration of urban areas with their congested roads and industrial belts. Source apportionment research from the Lombardy region (Pirovano et al. 2015) indicates that the major sources of PM_2.5_ include residential heating (e.g., fuel), transport, agriculture, background (including natural-source sand long-range transport), and other (including stationary industrial sources). The second is the location in the orographic “bowl” of the Po Valley, an extension of flat river lands enclosed between the Alps and Apennines mountains, which causes the stagnation of pollutants due to low ventilation (Giulianelli et al. 2014).

These factors help to characterize the Po Valley’s peculiarity with respect to different European areas with comparable urban and industrial density levels (Eeftens et al. 2012). Moreover, in addition to the urbanized and industrial areas, the remainder of the valley presents an intensive agricultural activity. Local studies on emission sources highlight a varying composition of the final concentration values depending on the position of monitoring stations and with different sources acting as local or diffused ones (for instance having high emissions from traffic close to cities, while having background biomass burning diffused in the whole region) (Bigi and Ghermandi 2016; Larsen et al. 2012). Indeed, given the EU Ambient Air Quality Directives that sets the Air quality standards for the protection of health at 25 μg/m^3^ for the averaging period of a calendar year, the Po valley shows values consistently near or above the threshold. These values often range in the 25–30 μg/m^3^ interval with peaks of > 30 μg/m^3^, which in Europe are only matched in Southern Poland and other smaller Eastern European clusters (EEA 2019).

Compared to its overall representation in the population, Lombardy is disproportionately impacted by COVID-19 related mortality, with approximately 53% of Italy’s COVID-19 deaths as of April 15, 2020 (Odone et al. 2020). Lombardy is also the most impacted Italian region as far as the total number of deaths in excess in the first quarter of 2020 compared to the same period of the previous years. Comparing the official COVID-19 death data with registry deaths, it emerges that the latter is almost 70% larger than the former in Lombardy, 27% larger in Emilia-Romagna and 18% and 16% in Veneto and Piemonte, respectively. It is, therefore, imperative to consider the role that PM may have played in such disproportionate COVID-19 deaths in Northern Italy.

There are a number of plausible pathways by which airborne PM may impact COVID-19 related morbidity and mortality. Existing data already finds a strong positive correlation between viral respiratory infection incidence and ambient PM concentrations (Ciencewicki and Jaspers 2007; Sedlmaier et al. 2009). One plausible pathway for this phenomenon is the fate and transport of the virus itself within the environment. A recent position paper by the Italian Society of Environmental Medicine argues that PM may act as both a carrier and substrate of the virus and thus influence the virus’ fate and transport in the environment and reaching susceptible receptors (Setti et al. 2020). Another pathway is the increase in susceptibility to COVID-19 mortality caused by long term exposure to PM. Fine PM is already known to affect cardiovascular and respiratory morbidity and mortality (Cakmak et al. 2018; Jeong et al. 2017; McGuinn et al. 2017; Yin et al. 2017). Moreover, among 1596 Italian COVID-19 patients who died in the hospitals, and for whom it was possible to analyze clinic charts, data showed substantial comorbidities including ischemic heart disease (27.9%); atrial fibrillation (22.4%); heart failure (15.6%); stroke (10.9%); hypertension (70.6%), and chronic obstructive pulmonary disease (17.9%) (Istituto Superiore di Sanità 2020). Biologically, long-term PM exposure may be responsible for a chronic inflammation status that causes the hyper-activation of the immune system and the life-threatening respiratory disorders caused by COVID-19 (Shi et al. 2020).

Some preliminary evidence is now emerging about COVID-19 that shows a positive relationship between air pollution and morbidity and mortality. Beyond qualitatively describing the European Air Quality Index for Northern Italy to argue the causal role of air pollution and the relatively high COVID-19 mortality observed in that region, Conticini et al. (2020) review the most recent existing toxicological and epidemiological literature. Based on existing evidence from other empirical studies, they clarify the relationship between air pollution, prolonged inflammation and immune system hyper-activation and immune suppression, and the link between the latter and acute respiratory distress syndrome, and respiratory mortality. Their paper is important in that it suggests a clinical and biologically plausible explanation to our analysis, but does not provide statistical evidence in support of the hypothesis. A separate empirical analysis by Becchetti et al. (2020) finds preliminary evidence that confirms such a positive effect of air pollution on mortality in Italy based on the analysis of death data at the province level. Similarly, Wu et al. (2020) show a positive association between long term PM exposure and COVID-19 related deaths in US counties. Ogen (2020) recently analysed data from 66 administrative regions in France, Spain, Italy, and Germany, and found that the highest COVID-19 deaths in these regions were associated with five regions of Northern Italy that also corresponded with the highest levels of atmospheric nitrogen dioxide (NO_2_). Cole et al. (2020) estimate the same relationship using Netherlands municipality data and find PM_2.5_ positively associated with COVID-19 cases, hospitalization, and deaths.

In this paper, we follow this emerging stream of the empirical literature and test the hypothesis that a higher average long-term exposure to PM_2.5_ is positively associated with the current extraordinarily high death toll in Northern Italy. We decided to focus on PM_2.5_ because, given the complexity of air pollution, it is quite common in air pollution epidemiology studies to focus the analysis on a single pollutant (Wu et al. 2020), although multipollutant analyses are certainly warranted. We selected PM_2.5_ for a variety of important reasons, including policy implications and evidence in the literature in terms of chronic health effects. Regarding its policy implications, we selected PM_2.5_ as opposed to PM_10_ because the former is more correlated with human activities than the latter, and it correlates with stronger health effects than PM_10_ does. With respect to respiratory mortality effects from the existing air pollution literature, the most robust evidence points to PM_2.5_ as opposed to other gaseous air pollutants (Bowe et al. 2019).

Mortality data are collected at the municipality level for the period January-April 2020. Given that mortality data are not disaggregated by mortality cause, death counts are measured as the difference from the last five-years mean to reflect the abnormal number of deaths caused by the spreading of the pandemic. Since PM_2.5_ can be associated to generic mortality even in the absence of the pandemic outbreak (Dominici et al. 2003; Katsouyanni et al. 2001; Samet et al. 2000), we also estimate the impact of PM_2.5_ on the excess mortality in the sample using 2019 data, a time in which the coronavirus epidemic had presumably not yet begun. Data on PM_2.5_ concentration at the municipality level refer to the years prior 2020 to account for long-term population exposure. We assign municipality PM_2.5_ concentration by a set of different methods of spatial interpolation (kriging) of monitoring station data related to the years 2015–2019.

We estimate a negative binomial model of excessive deaths on historical PM_2.5_ concentrations and a series of control variables that may plausibly affect both PM_2.5_ concentration and mortality, including population density; the spatial concentration of the industrial manufacturing sites; climatic conditions observed during the first quarter of 2020; and the demographic composition of the municipal population among others. In addition, we consider spatial heterogeneity in the distribution of the number of deaths related to regional and local unobservable factors. We account for region-specific effects because regions, in Italy, are the administrative units in charge of managing the health systems and the measures taken to trace and contrast the spreading of the pandemic varied greatly among even contiguous regions. We also account for local effects common to functionally linked clusters of municipalities (the Local Labour Systems—LLS). We deem this part of the identification strategy crucial because the relationship between PM_2.5_ and COVID-19 mortality may be confounded by several other factors, some of which were not observable or measurable, but are nevertheless intrinsically related to the geographical location of the observed units.

The remainder of the paper is organized as follows. The next section introduces the empirical strategy and describes the dataset. The results are presented and discussed in section three, considering the total number of (excess) deaths. Section four draws the conclusions and highlights the limitation of the study and the indications for future research.

## Empirical Strategy and Data

**Fig. 1:**
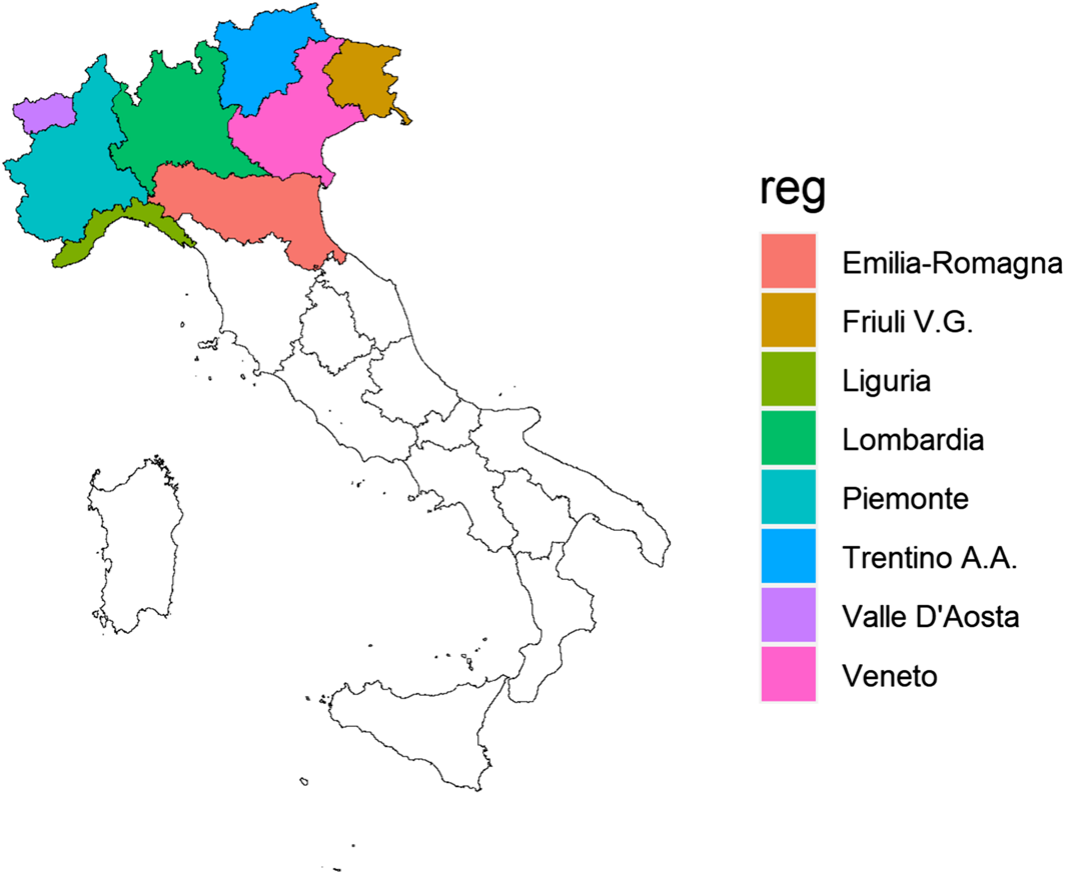
Italian regions included in the study

Our analysis is restricted to the study area of Northern Italy (Fig. 1), which encompasses the sub-regions of Valle D’Aosta, Piemonte, Liguria, Lombardia, Emilia-Romagna, Veneto, Friuli-Venezia Giulia and Trentino-Alto Adige/Südtirol. Official territorial data on COVID-19 mortality in Italy are available at the rather aggregate regional or provincial level, corresponding to the levels 2 and 3, respectively of the European nomenclature units for territorial statistics (NUTS).^5^ In addition, these official data refer to the deaths of patients tested positive for severe acute respiratory syndrome coronavirus 2 (SARS-CoV2) only and do not include (potential) patients without COVID-19 diagnosis because they were not tested and died at home or elsewhere. Hence, the officially reported deaths are likely underestimated. Because testing policies vary among regions in Italy, the induced measurement error is also non-randomly distributed among the provinces. Ciminelli and Garcia-mandicó (2020) compare the official COVID-19 fatality rates with historical death data and report that deaths were higher than official fatalities throughout the period of COVID-19 epidemic.

**Fig. 2:**
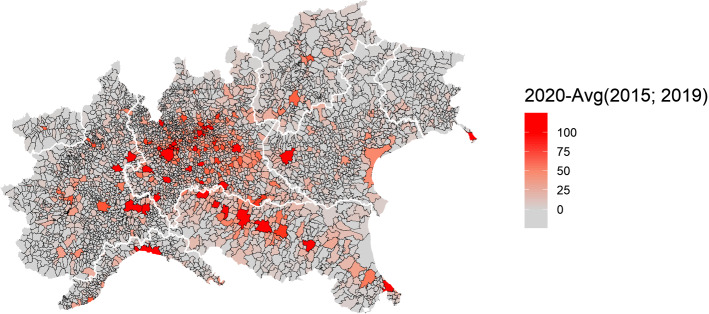
Spatial distribution of cumulative excess deaths in sample municipalities, Northern Italy, January 1—April 30, 2020

Working under the assumption that COVID-19 deaths are underestimated in Italy, the choice is made in this paper to use the total deaths from the official registries, accordingly, and to scale the analysis at the municipality level, the smallest administrative units, to have a more granular representation of the spatial dimension of the phenomenon. Since we are interested in excess deaths, we take the difference between the number of deaths in the period January 1—April 30, 2020, and the average number of deaths in the same period of the previous 5 years (*ExDeaths*) and use this metric as the dependent variable in our statistical model. Figure 2 displays the geographical distribution of the above-described data among the 4041 municipalities for which data is available.

The variable is assumed to follow a Negative Binomial distribution, a generalization of the Poisson distribution that avoids the restrictive mean–variance equality of the latter, and is modelled as follows:

1 $$\begin{aligned} & {\text{ExDeaths}}_{i} \sim NB\left( {\mu_{i} ,\theta } \right) \\ & \log (\mu_{i} ) = \alpha + \beta PM_{i} + \delta^{\prime }X_{i} + \varepsilon_{i} \\ \end{aligned}$$

where $\theta$ is the overdispersion parameter to be estimated and $\mu_{i}$ is the municipality-specific expectation conditional on the value of the covariates. Among the covariates, *PM* is the concentration of fine particulate matter in municipality *i* and $\beta$ is the associated parameter, which we expect positive and statistically different from zero; *X* is a vector of control variables that adjusts for the potential confounding effects and includes the (log of) total population as the offset while $\varepsilon$ is a normally-distributed error term.

Our main source of PM_2.5_ data is the European Environmental Agency’s (EEA) air monitoring database, which is provided to EEA by the Institute for Environmental Protection and Research (ISPRA). ISPRA conducts ground-level air measurements of PM_2.5_ air concentrations (µg/m^3^) collected at 268 monitoring sites throughout Italy. Specifically, we use the EEA’s E1a and E2a datasets, which are primary validated assessment data and primary up-to-date assessment data reported by the European Member States, respectively. Although the measurements come both in hourly and daily averaging formats, we work with daily values and use them to obtain yearly aggregates for the years 2015, 2016, 2017, 2018, and 2019. However, because model (1) does not include a time component, we further compute a six-year averaging time to obtain a metric of long-term (chronic) PM_2.5_ concentration levels throughout different spatial units of Northern Italy. The number of 6 years for the reference period is sufficiently long to account for long-term exposure while being not too long to be affected by the mobility of people among municipalities, and it is in line with existing literature assessing long-term effects of PM exposure (Yorifuji et al. 2019). Since the air monitoring stations provide only partial spatial coverage for municipality-level PM_2.5_ concentration data, we impute missing observations using a spatial interpolation model. Specifically, we fill in the gaps using a mean stationary Ordinary Kriging (see Bivand et al. 2013, p 209) defined through an exponential covariance function with nugget, partial sill and range parameters estimated through (restricted) maximum likelihood methods. Figure 2 displays the resulting PM_2.5_ concentration data.^6^

**Fig. 3:**
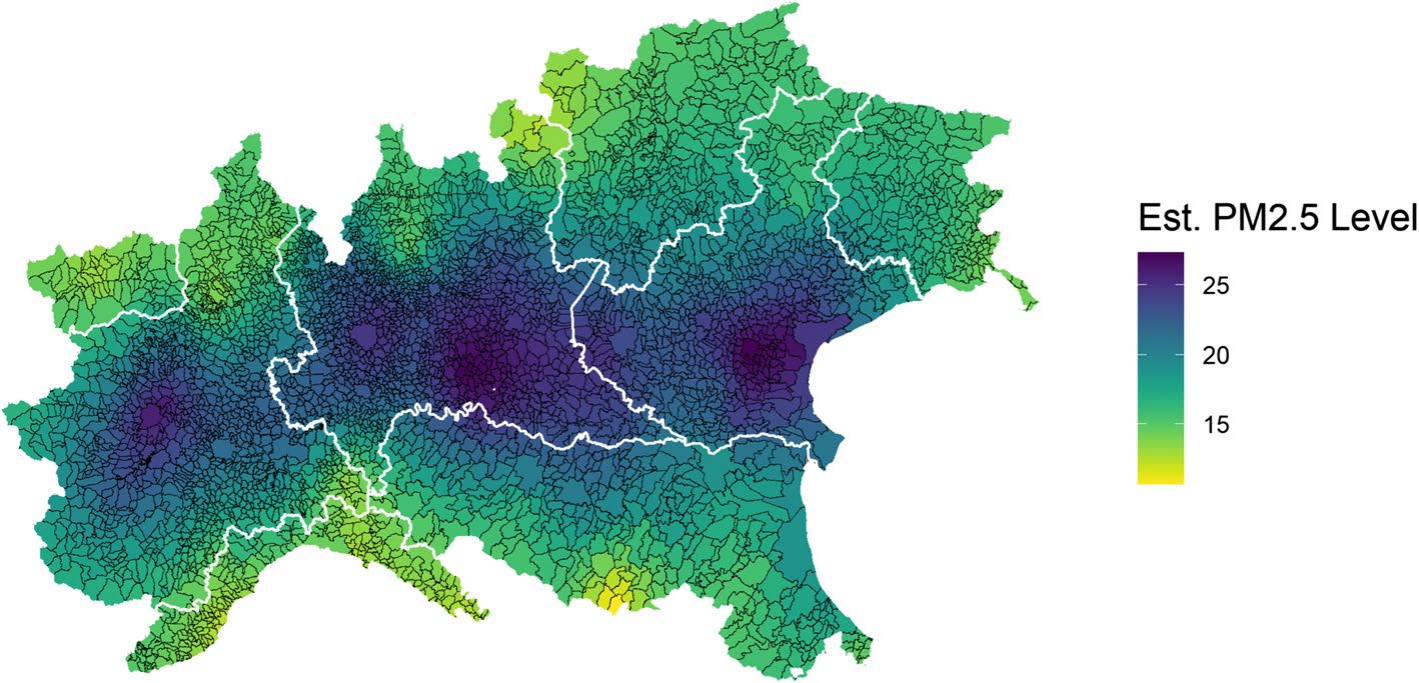
Spatial distribution of PM2.5 concentration levels in the sample municipalities, simple kriging of monitoring stations, average across years 2015–2019

Comparing Figs. 2 and 3, it is possible to visually appreciate a spatial coincidence between higher levels of excess mortality and higher levels of PM_2.5_, in particular in the Lombardia region which notably is the region with both the highest particulate concentration and the highest number of excess mortality.

The hypothesis that PM_2.5_ concentration affected COVID deaths, that is $\hat{\beta } > 0$, is tested among several possible specifications. In model (2) we include regional effects $\left( {\lambda_{j} } \right)$. These effects are expected to capture the aspects related to the management of the outbreak, which may have systematically influenced COVID-19 mortality and that are common to all the municipalities in the same region. Italy has a national health system that ensures equal access to healthcare to all citizens. The system is managed by regions at the local level, and, in the specific case of this pandemic, regions were responsible for defining the testing and contact-tracing protocols and implementing the necessary measures to contain the outbreak, among which the measure to protect healthcare workers. In model (3), we include LLS-specific effects $\left( {e_{k} } \right)$. LLS are spatial clusters of contiguous municipalities related by commuting flows that share a common specialization in a specific sector of manufacturing production and correspond to the conceptualization of Marshallian districts (Becattini 2002). The number of LLS clusters per-region and the total number of municipalities belonging to clusters are reported in Table 1, along with the minimum, maximum, and average cluster size.

**Table 1:** Number of LLS spatial clusters in each region

Region	N. LLS	N. of municipalities	Smallest LLS (N. of municipalities)	Largest LLS (N. of municipalities)	Average number of municipalities by LLS
Emilia-Romagna	42	328	1	38	8
Friuli-Venezia Giulia	13	215	1	51	16
Liguria	17	234	1	26	12
Lombardia	57	1507	1	174	25
Piemonte	39	1181	1	104	26
Trentino-Alto Adige/Südtirol	27	291	1	30	10
Valle d’Aosta	5	74	3	29	12
Veneto	49	563	1	52	11

The use of LLS captures the interlinkages within neighbouring municipalities that may have favoured the geographical spreading of coronavirus around specific hotspots. Mortality data are then expected to vary among municipalities in different LLS, but differences are expected to be non-systematic in this case. In model (4) we include both the regional fixed effects and the LLS random effects.

2 $$\begin{aligned} & ExDeaths_{i} \sim NB\left( {\mu_{ij} ,\theta } \right) \\ & \log (\mu_{ij} ) = \alpha + \beta PM_{ij} + \delta^{\prime }X_{ij} + \lambda_{j} + \varepsilon_{ij} \\ \end{aligned}$$

3 $$\begin{aligned} & ExDeaths_{i} \sim NB\left( {\mu_{i} ,\theta } \right) \\ & \log (\mu_{ik} ) = \alpha + \beta PM_{ik} + \delta^{\prime }X_{ik} + u_{ik} \\ & u_{ik} = \varepsilon_{ik} + e_{k} \\ \end{aligned}$$

4 $$\begin{aligned} & ExDeaths_{i} \sim NB\left( {\mu_{ijk} ,\theta } \right) \\ & \log (\mu_{ijk} ) = \alpha + \beta PM_{ijk} + \delta^{\prime }X_{ijk} + \lambda_{j} + u_{ijk} \\ & u_{ijk} = \varepsilon_{ijk} + e_{k} \\ \end{aligned}$$

Control variables to be included in the model were chosen to avoid any potential spatial confounding effect and considering as well the emerging literature on the impact of PM on COVID-19 related deaths (Cole et al. 2020; Wu et al. 2020). The population density and per-capita income account for urbanisation level. The most densely populated and wealthy municipalities are among the most polluted due to the spatial concentration of manufacturing and service activities but are also the places where the contagion could have been easier, with a potential impact on mortality. In addition to the density of population, the shares of municipality area occupied by industrial sites and the average size of manufacturing firms are included in the regression because they are related to pollutant concentration and possibly to mortality. National measures to stop the spreading of the viral infection (lockdown) involved the service sector to the largest extent while many manufacturing activities, being considered necessary, were left open and, in the absence of social distance and individual protection measures, the geographical concentration of these activities in a municipality with their complex logistics and transport interconnections, and the size of plants, may have influenced mortality. Average temperature, for which an association with COVID-19 deaths has also been found (Ma et al. 2020), is also included in the regression.^7^ Moreover, COVID-19 incidence has proven to be higher among men than women and people aged 65 or more. Hence these two variables are considered in the model, even though these aspects are not necessarily connected with the average PM_2.5_ exposure in a municipality. Underlying socioeconomic conditions can also play a role in COVID-19 related mortality (Goutte et al. 2020). Brandt et al. (2020) and Mukherji (n.d.) have shown that, in the US, COVID-19 is more threatening for ethnic minorities, and we believe that the share of migrants, identified as non-EU citizens, can control for this aspect influencing the observed excess mortality. On the other hand, Mukherji (2020) and Goutte et al. (2020) also find that places with a higher share of the population with a low level of education have higher deaths. In our paper, given the lack of updated data on education at the municipal level, we proxy it with the percentage of university students on the total population. The distance from the closest airport is a proxy for the functional and relational linkage between a municipality and a place of highly frequent national and international connections and potential sources of coronavirus spreading. Finally, we consider the number of hospital beds as a proxy for the supply of health services to account for the fact that many people died at home without being diagnosed for coronavirus due to the shortage of beds in public structures. The full details of the variables in the model, including sources and summary statistics, are presented in Table 2.

**Table 2:** Description of model variables and summary sample statistics

Variable	Description	Mean	Median	SD
ExDeaths	Number of deaths in the period January 1—April 30 2020—absolute difference compared to the average of the past five years, source: ISTAT	9.32	2	37.13
PM_2.5_	Fine (2.5 µg/m^3^) particulate matter concentration obtained by spatial interpolation of monitoring stations, average across the years 2015–2019, source: European Environmental Agency	19.67	20.85	4.15
Pop. density	Population density computed as total population in number of inhabitants on January 1 2020 over the total artificial area in Km^2^, sources: ISTAT and European Environmental Agency—Corine land Cover data	34.44	14.69	58.19
PC income	Average per-capita income, source: Ministry of Finance, 2019	15,658.46	15,564.52	2497.44
% Ind. Land	Share of industrial area on total municipality surface measures through satellite observation, source: European Environmental Agency—Corine land Cover data	2.62	0.02	5.17
% Small Ent	Share of enterprises with less than 10 employees, source: Registro statistico delle Unità Locali (ASIA—UL)	94.26	94.44	3.80
Temperature	Average mean skin temperature during the death observation period, source: Copernicus ERA5 0.25° × 0.25° grid resolution dataset.	3.75	5.34	4.01
Female/Male	Ratio between female and male population, source: ISTAT	1.01	1.02	0.06
% Over 65	Share of population older than 65, source: ISTAT	23.36	22.72	4.93
% non-EU	Share of non-EU residents, source ISTAT	1.88	1.51	1.55
% Univ. Stud.	Definition, source: share of University students over total population, source: Ministry of University and Research	82.28	19.49	289.57
Dist. Airport	Distance in meters to the closest Airport, source: our computation based on European Environmental Agency—Corine land Cover data	23,255	21,437.22	12,823.72
PC Hospital Beds	Number per-capita hospital beds in the municipality, source: Health Ministry	0.001	0.00	0.012
Population	Total population, source: ISTAT	6710.32	2549	38,814.47

Having accounted for the confounding effect due to the omission of relevant information from the empirical specification, we exclude any other potential source of endogeneity considered in similar papers. In particular, we exclude endogeneity due to measurement error in the outcome variable and the main independent variable. Concerning the outcome variable, the relationship between deaths and cases with fine PM could be spurious because more cases could be registered, and more individuals tested in highly polluted areas as people there are more likely to show COVID-19 symptoms due to the chronic inflammation induced by PM. The high toll of deaths of people diagnosed with COVID-19 would be a natural consequence of that. In contrast, the number of deaths in excess, used in this paper, is not affected by testing problems since it considers all the potential COVID-19 deaths. Concerning the PM variable, measurement errors are likely to occur when using satellite data or modelled data. We preferred to use PM_2.5_ levels observed from monitoring stations to avoid such a measurement error. Some caution is needed in the spatial interpolation because the method chosen to fill the missing data may underestimate the value in locations farther from the monitoring stations. With this concern in mind, we test the robustness of our results using PM_2.5_ data obtained from different interpolation approaches.

## Results

As indicated in Table 2, the overall average of PM2.5 for the study area between 2015 and 2019 is roughly 20 µg/m^3^, as most municipalities in Norther Italian regions belong to industrial and agricultural intensive locations. The average mortality between 2015 and 2019 for the period of interest (January 1—April 30) was 25 deaths, while it grew to 34 in 2020. That results in an average excess death of 9, with standard deviation four times as larger (see Table 2).

Estimation results from the negative binomial models are summarised in Table 3 for the four different specifications of the model (1—no geographical effects; 2—regional fixed effects; 3—LLS random effects; 4—regional fixed effects and LLS random effects). In the lower part of the table, the estimated overdispersion parameter, the Akaike Information Criterion (AIC), and the Moran’s test for the null hypothesis of absence of spatial autocorrelation^8^ in the residuals (Moran 1950) are reported.

**Table 3:** Estimation result of main regressions, dependent variable: excess deaths during the period January 1—April 30 2020, municipalities in Northern Italy

	Model (1)		Model (2)		Model (3)		Model (4)	
	Estimate	(SE)	Estimate	(SE)	Estimate	(SE)	Estimate	(SE)
Intercept	− 6.314***	(1.834)	− 6.862***	(1.717)	− 5.369**	(1.844)	− 6.254***	(1.807)
PM_2.5_	0.128***	(0.008)	0.085***	(0.009)	0.089***	(0.014)	0.089***	(0.014)
Female/Male	− 1.451**	(0.449)	− 0.726	(0.427)	0.213	(0.426)	0.180	(0.422)
% Over 65	0.076***	(0.006)	0.074***	(0.006)	0.066***	(0.006)	0.065***	(0.006)
Temperature	− 0.064***	(0.007)	− 0.046***	(0.007)	− 0.048***	(0.011)	− 0.040***	(0.010)
Pop. density	− 0.011	(0.030)	− 0.099***	(0.028)	− 0.005	(0.029)	− 0.016	(0.028)
% Ind. Land	− 0.009	(0.006)	− 0.009	(0.005)	− 0.008	(0.005)	− 0.008	(0.005)
% Small Ent	− 0.008	(0.007)	− 0.017*	(0.007)	− 0.009	(0.007)	− 0.011	(0.007)
PC Income	− 0.199	(0.166)	− 0.082	(0.157)	− 0.385*	(0.173)	− 0.270	(0.170)
% non-EU	0.015	(0.016)	0.020	(0.015)	− 0.018	(0.016)	− 0.013	(0.015)
% Univ. Stud.	0.000	(0.000)	0.000	(0.000)	0.000	(0.000)	0.000	(0.000)
PC Hospital Beds	0.418	(2.094)	− 1.001	(2.056)	− 0.517	(1.771)	− 0.746	(1.769)
Dist. Airport	− 0.159***	(0.028)	− 0.091***	(0.026)	− 0.087*	(0.039)	− 0.068	(0.035)
	*Regional fixed effects*							
Lombardia			0.784***	(0.081)			0.598***	(0.139)
Emilia-Romagna			0.185*	(0.094)			− 0.013	(0.147)
Piemonte			− 0.024	(0.080)			− 0.034	(0.136)
Veneto			− 0.823***	(0.097)			− 0.894***	(0.149)
theta	0.571		0.69		0.894		0.894	
Observations	4041		4041		4041		4041	
AIC	21,045		205,598		20,397		20,297	
log-Likelihood	− 10,509		− 10,281		− 10,183		− 10,129	
Moran’s I Test [*p* value in parenthesis]	0.276 [< 0.001]		0.143 [< 0.001]		0.005 [0.784]		0.001 [0.596]	

***p < 0.01, **p < 0.05, *p < 0.1

The four specifications provide consistent results in terms of the direction and significance of PM_2.5_ coefficients. The overall effect of PM_2.5_ on COVID-19-related excess mortality is positive and statistically significant in all models. The estimated incidence rate ratios, reported in Table 4 with their confidence interval, for Model 1, 2, 3 and 4 are 13.7%, 8.9%, 9.3%, and 9.3%, respectively.

**Table 4:** Marginal effects of an increase in PM2.5 concentration on excess deaths in Northern Italy during COVID-19 outbreak

	Estimate	2.50%	97.50%
Model (1): No territorial effect	1.137	1.119	1.154
Model (2): Regional FE	1.089	1.069	1.109
Model (3): LLS RE	1.093	1.064	1.122
Model (4): Regional FE and LLS RE	1.093	1.063	1.123

In model 2, the regional fixed effects coefficients are statistically significant. They indicate that other things being equal, the number of deaths in municipalities in Lombardy and Emilia Romagna has been systematically higher compared to base category^9^ and in municipalities in Veneto it has been systematically lower. The significance of the coefficient for Emilia Romagna, however, drops after including the random effects in the model. Since the first three models are nested into model 4 it is also possible to compare the models in terms of AIC. Model 4 performs substantially better than the other three. In general, the inclusion of RE in models 3 and 4 leads to a decrease in the value of the AIC. In models 1 and 2 the residuals appear spatially autocorrelated, as the null hypothesis of no spatial autocorrelation is rejected in both cases (*p* < 0.001). The introduction of the LLS random effects appears to solve the issue of autocorrelation.

**Fig. 4:**
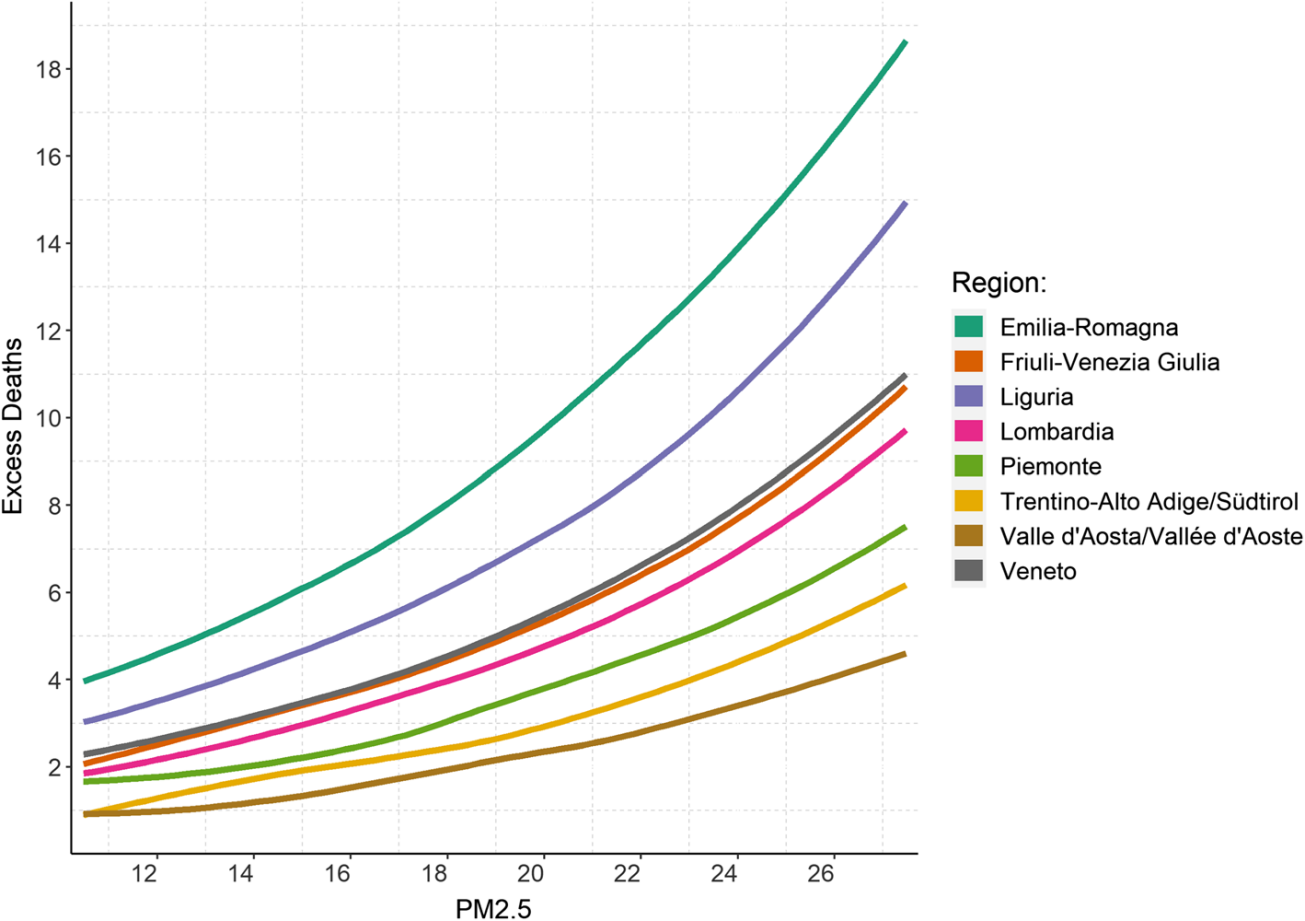
Expected excess deaths in the average municipality against the observed value of PM_2.5_, by region

Based on the estimates of model 4, we compute the expected value of excess deaths conditional on covariates (taken at the average level) in the average city for varying levels of PM_2.5_ and show how the expected number of deaths by region varies at different concentration levels in Fig. 4. Notably, Emilia–Romagna and Liguria are the regions in which a a reduction of average fine PM from the highest level to the lowest would have benefited the most.

### Robustness checks

**Fig. 5:**
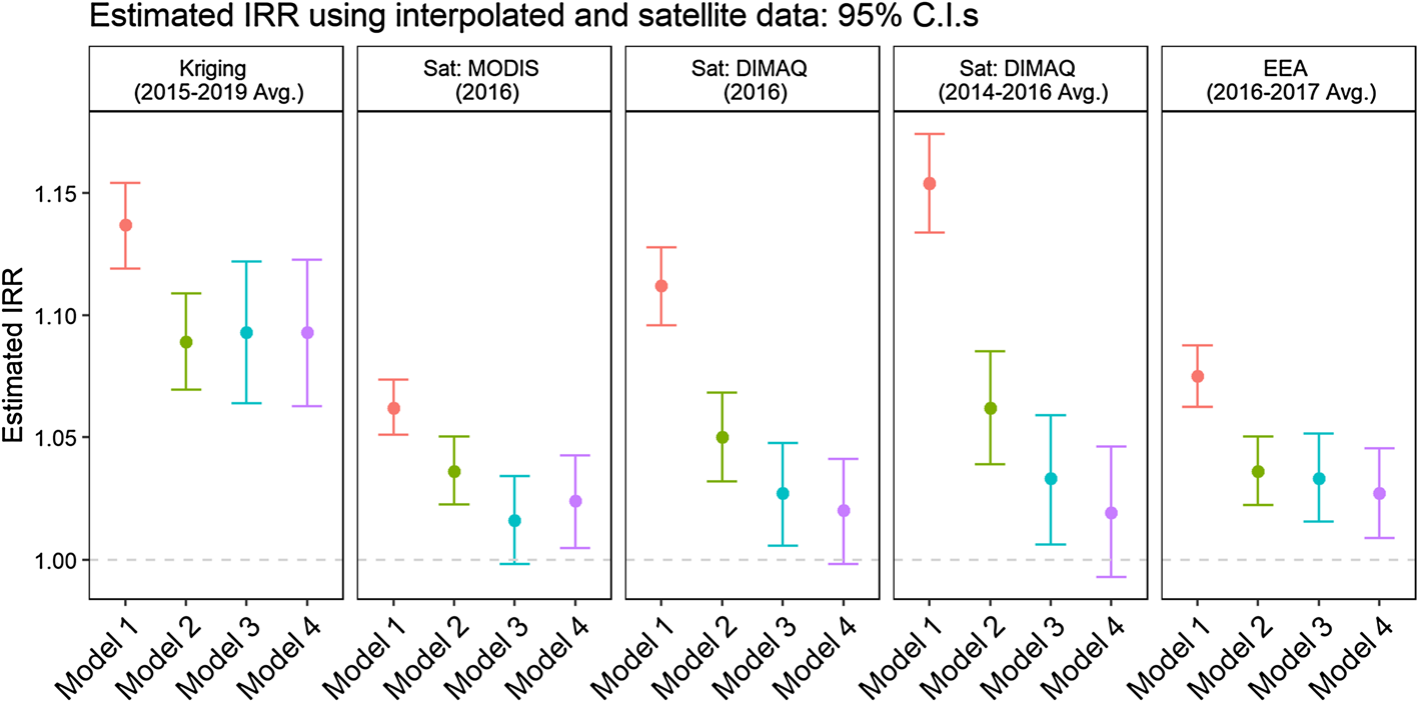
Robustness check: estimated IRR (PM variable only) for models (1)–(4) using spatially interpolated data and four alternative satellite measures of particulate concentration

For robustness check of the PM_2.5_ metric used in our study, we explored the influence that other alternate PM_2.5_ metrics may have on the direction and magnitude of the observed associations. Figure 5 depicts the point estimates and the 95% confidence interval for the Incidence Rate Ratios (IRR).^10^ We find that while data from satellite elaborations (MODIS^11^ and DIMAQ^12^), and monitoring stations’ interpolation EEA^13^ PM_2.5_ models result in IRRs trending in the same direction, the point estimates for IRRs are lower than our primary analysis which was based on a combination of ground monitoring and kriging. The lower IRR point estimates are unsurprising because the underlying data for the alternate PM_2.5_ metrics do not have the same temporal coverage as the ground-level monitoring data (2015–2019). This lack of temporal coverage contributes to non-differential exposure misclassification, which, in turn, would lead to suppressing effect estimates towards the null. Despite this, it is encouraging to find that regardless of the PM_2.5_ metric used, the direction of the observed associations remains, and so does statistical significance.

**Fig. 6:**
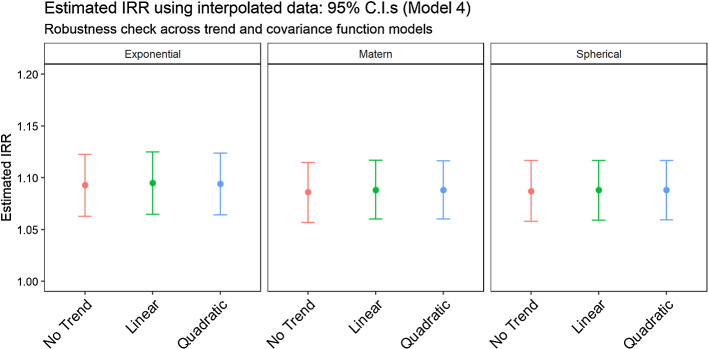
Robustness Check: estimated IRR (PM variable only) for PM in Model 4 using three different covariance functions and three alternative trend models

As previously anticipated, we re-estimate model (4) using different specifications of the Kriging interpolator. In particular, we first relax the mean-stationarity assumption of Ordinary Kriging by modelling the mean function of the process through both a linear and a quadratic trend in latitude and longitude. Next, we replace the simple Exponential function with a Spherical model and a more flexible Matérn kernel with the characteristic parameter set at 3/2 (to preserve mean-square differentiability). All these specifications still assume covariance stationarity. Figure 6 and Table 5 in the Appendix report the estimated Incidence Rate Ratios (IRR) regression coefficients for the PM variable in model (4) under these multiple setups: both point estimates and 95% confidence intervals indicate that there are no substantive differences between using different trend or covariance models, indicating that our result is robust to alternative specification of the interpolation method.

**Table 5:** Estimated regression coefficient (PM variable only) for Model 4 using nine Kriging specifications: three different covariance functions (Exponential, Matérn and Spherical) time three alternative trend models (constant trend, linear trend and quadratic trend)

Method	Covariance	Trend	Estimate	Std. Err.	*p* value	AIC
Kriging	Exponential	No Trend	0.089	0.014	< 0.001	20,296
		Linear	0.091	0.014	< 0.001	20,295
		Quadratic	0.09	0.014	< 0.001	20,295
	Matern	No Trend	0.082	0.014	< 0.001	20,300
		Linear	0.085	0.013	< 0.001	20,296
		Quadratic	0.085	0.013	< 0.001	20,295
	Spherical	No Trend	0.083	0.014	< 0.001	20,300
		Linear	0.084	0.014	< 0.001	20,298
		Quadratic	0.084	0.014	< 0.001	20,298
Satellite	MODIS 2016	–	0.02	0.01	0.013	20,329
	DIMAQ 2016	–	0.02	0.01	0.068	20,331
	DIMAQ 2014–2016	–	0.02	0.013	0.148	20,333
EEA 2016–2017		–	0.026	0.01	0.003	20,326

The table also includes the estimated regression parameters for Model (4) using Satellite and EEA data

## Discussion

In each of the four specifications presented, the coefficient related to PM_2.5_ is always of the hypothesized direction and statistically different from zero. Precisely and consistently with previous results for the original SARS-Coronavirus during the 2003 outbreak (Cui et al. 2003), an increase in air pollution exposure is associated with increased mortality for COVID-19. The first panel in Fig. 4, as well as Table 4, suggests that, when using interpolated data from ISPRA monitoring stations, the increase in mortality rate due to a one-unit increase in PM_2.5_ concentration varies between 14% (model 1—highest rate) and nearly 9% (model 4—lowest rate). The 95% confidence interval for the point estimate in model 4 lies between roughly 6% and 12%. Our findings fall in line with both Wu et al. (2020) and Cole et al. (2020) papers. Specifically, both papers find a positive ecological relationship between PM2.5 and COVID-19 mortality. In relation to a 1 µg/m3 increase in PM2.5, Wu et al. find 8% change in COVID-19 mortality, Cole et al. find the same increase associated to additional 3 COVID-19 deaths (almost 17% if compared to their sample mean), and our paper finds 9% increase in COVID-19 related excess mortality. Despite this similarity in results, the two key differences between our study and the others relate to the exposure assessment method and the outcome assessment method. In our study we use a spatial interpolation method (kriging) from ground-level monitoring data, whereas these other two studies utilize PM2.5 gridded surfaces such as chemical transport modelling in the case of Cole et al. and a hybrid approach using chemical transport, aerosol optical depth and land use regression modelling in the case of Wu et al. With respect to COVID-19 mortality data, Wu et al. use county-level data from the Johns Hopkins University, Center for Systems Science and Engineering Coronavirus Resource Center, which is comprised of COVID-19 deaths tabulated by the US Centers for Disease Control and Prevention and State health departments. In Cole et al., researchers obtained COVID-19 deaths by residential address and aggregated these to the municipality level. The obvious difference between their study and ours is that we used a surrogate excess mortality measure due to the issues of reliability for COVID-19 death data, as we have already discussed. The other relevant difference between our study and the Wu et al. and Cole et al. studies is that we subsample the total cohort of Italian municipalities to only regions with a very high mortality rate, which are also the regions most affected by the air quality problems. On the other hand, when satellite data are used, our estimate yields lower incidence ratios. Although ground-level concentration metrics come with fewer measurement errors, satellite data proves nevertheless useful in corroborating both the direction and the significance of the effect of interest. This redundancy is particularly relevant in light of the relatively few stations capable of detecting the finest particulate.

With reference to model (4) and the remaining covariates, we observe no effect related to population density or income or the extent of industrial areas in the municipality. Likewise, there is no evidence suggesting significant links between the share of non-EU residents, the female to male ratio (which disappears after we incorporate the random effects), and the level of education (proxy by the percentage of university students) on the dependent variable of interest. On the other hand, our results suggest a negative association between temperatures and mortality due to COVID-19. Finally, as expected, we find that municipalities with higher shares of the population aged 65 or more have been most affected. The distance from the closest airport, a measure of relational connectedness that also proxy for the exposure to the contagion process, deserves a last comment. We find that municipalities closer to an airport experience a higher number of deaths in excess. We speculate that the result could be related to a higher likelihood for these municipalities to become clusters of contagion in the initial phase of the pandemic, but a causal link cannot be inferred based on our result ad the topic needs more research to be addressed adequately.

We conclude our analysis by checking the consistency of our results to different choices of the dependent variable. Existing evidence (Dominici et al. 2003; Pascal et al. 2014; Samet et al. 2000; Yin et al. 2017) associates fine PM to severe cardiovascular and respiratory diseases and mortality. In European cities, in particular, an estimated increase in the number of daily deaths of 0.7% is associated with an increase of 10 µg/m^3^ of PM_10_ (Katsouyanni et al. 2001). This evidence suggests that long term PM exposure may have had an overall effect on deaths even before the outbreak in the sample municipalities, making it more difficult to isolate the real effect on COVID-19 deaths. We thus run model (4) using the total number of deaths in the same observation period of 2019 as the dependent variable to understand whether the effect of fine PM_2.5_ on mortality has been more severe during the pandemic. We find no evidence of an effect of PM_2.5_ on total deaths for 2019 in the sampled municipalities, suggesting that the effect of PM exposure on the mortality rate is closely connected to the novel coronavirus outbreak (see Table 6 in the Appendix). However, since the dependent variable in this “placebo” regression cannot be directly compared to the excess mortality, we repeat the test using total mortality for the year 2020. Although the latter includes both COVID-19 related and unrelated deaths, these two variables represent data generating processes of the same nature. As expected, both the regression coefficients and IRRs calculated regressing total deaths in 2020 suggest a positive and statistically significant effect of exposure to fine particulate on mortality, even though its magnitude is greatly reduced if compared to the estimates in Tables 3 and 4 (see Table 7 in the Appendix). Presumably, the effect of PM_2.5_ concentration on COVID-19 related mortality becomes muted by the noise introduced when accounting for other causes of death. This would also explain the non-significant PM_2.5_ coefficient in the first “placebo” regression.

**Table 6:** Estimation results for the placebo regression, dependent variable: total number of deaths during the period Jan1-April 30 2019, municipalities in Northern Italy

	Model (1)		Model (2)		Model (3)		Model (4)	
	Estimate	(SE)	Estimate	(SE)	Estimate	(SE)	Estimate	(SE)
Intercept	− 5.072***	(0.806)	− 5.209***	(0.760)	− 5.530***	(0.812)	− 5.809***	(0.790)
PM_2.5_	0.044***	(0.003)	0.034***	(0.004)	0.033***	(0.005)	0.031***	(0.006)
Female/Male	− 0.034	(0.202)	0.304	(0.193)	0.669***	(0.193)	0.642***	(0.191)
% Over 65	0.062***	(0.003)	0.061***	(0.003)	0.057***	(0.003)	0.057***	(0.003)
Temperature	− 0.018***	(0.003)	− 0.013***	(0.003)	− 0.014**	(0.004)	− 0.010*	(0.004)
Pop. Density	− 0.010	(0.013)	− 0.048***	(0.012)	− 0.001	(0.012)	− 0.008	(0.012)
% Ind. Land	− 0.004	(0.003)	− 0.004	(0.002)	− 0.004	(0.002)	− 0.004	(0.002)
% Samll Ent.	− 0.003	(0.003)	− 0.006	(0.003)	− 0.002	(0.003)	− 0.003	(0.003)
PC Income	− 0.215**	(0.073)	− 0.201**	(0.069)	− 0.217**	(0.076)	− 0.177*	(0.074)
% non-EU	0.006	(0.007)	0.009	(0.007)	− 0.002	(0.007)	0.000	(0.007)
% Univ. Stud.	0.000	(0.000)	0.000	(0.000)	0.000	(0.000)	0.000	(0.000)
PC Hospital Beds	0.723	(0.904)	0.231	(0.863)	0.364	(0.827)	0.223	(0.825)
Dist. Airport	− 0.040**	(0.012)	− 0.014	(0.011)	− 0.018	(0.016)	− 0.007	(0.014)
	*Regional fixed effects*							
Lombardia			0.317***	(0.036)			0.289***	(0.055)
Emila-Romagna			0.118**	(0.04)			0.072	(0.057)
Piemonte			− 0.026	(0.035)			− 0.023	(0.053)
Veneto			− 0.283***	(0.041)			− 0.274***	(0.057)
theta	3.29		3.92		4.87		4.89	
Observations	4041		4041		4041		4041	
AIC	28,517		28,119		27,971		27,869	
log-Likelihood	− 14,244		− 14,041		− 13,970		− 13,915	

***p < 0.01, **p < 0.05, *p < 0.1

**Table 7:** Estimation results for the placebo regression, dependent variable: total number of deaths during the period Jan1–April 30 2020, municipalities in Northern Italy

	Model (1)		Model (2)		Model (3)		Model (4)	
	Estimate	(SE)	Estimate	(SE)	Estimate	(SE)	Estimate	(SE)
Intercept	− 3.876***	(0.280)	− 3.748***	(0.277)	− 3.641***	(0.295)	− 3.667***	(0.292)
PM_2.5_	− 0.001	(0.001)	0.000	(0.001)	0.000	(0.002)	0.002	(0.002)
Female/Male	0.313***	(0.077)	0.325***	(0.076)	0.357***	(0.079)	0.354***	(0.079)
% Over 65	0.056***	(0.001)	0.054***	(0.001)	0.053***	(0.001)	0.053***	(0.001)
Temperature	0.002	(0.001)	0.001	(0.001)	0.001	(0.001)	0.000	(0.001)
Pop. Density	− 0.014***	(0.003)	− 0.012***	(0.003)	− 0.006	(0.003)	− 0.006	(0.003)
% Ind. Land	− 0.002*	(0.001)	− 0.002**	(0.001)	− 0.002**	(0.001)	− 0.002**	(0.001)
% Samll Ent.	0.001	(0.001)	0.000	(0.001)	0.001	(0.001)	0.000	(0.001)
PC Income	− 0.238***	(0.025)	− 0.247***	(0.026)	− 0.260***	(0.028)	− 0.258***	(0.028)
% non-EU	0.000	(0.002)	0.002	(0.002)	0.002	(0.003)	0.002	(0.003)
% Univ. Stud.	0.000	(0.000)	0.000	(0.000)	0.000	(0.000)	0.000	(0.000)
PC Hospital Beds	0.433	(0.365)	0.349	(0.362)	0.469	(0.354)	0.439	(0.355
Dist. Airport	0.016***	(0.004)	0.017***	(0.004)	0.017**	(0.005)	0.017***	(0.005)
	*Regional fixed effects*							
Lombardia			0.013	(0.013)			0.002	(0.019)
Emila-Romagna			0.047***	(0.013)			0.028	(0.019)
Piemonte			0.076***	(0.013)			0.071***	(0.018)
Veneto			− 0.035*	(0.014)			− 0.039*	(0.02)
theta	3.29		3.92		4.87		4.89	
Observations	4041		4041		4041		4041	
AIC	27,432		27,327		27,269		27,238	
log-Likelihood	− 13,702		− 13,645		− 13,619		− 13,600	

***p < 0.01, **p < 0.05, *p < 0.1

## Conclusion

Italy is among the countries most severely affected by the new coronavirus, with more than 230 thousand confirmed cases and more than 30 thousand deaths as of the end of May. Yet, the spatial distribution of confirmed cases and deaths suggest that the effects of the viral infection spreading largely vary across the regions of the country but also within regions. In this work, we examined the role of ambient PM_2.5_ in explaining the spatial variation in deaths that occurred throughout the most extreme time period of the epidemic. The results in the paper, that suggest a positive relationship between PM_2.5_ concentration and COVID-19 related excess mortality, are robust to different specifications PM_2.5_ and estimation strategies, even after controlling for additional confounder factors. Coherently with previous findings in the literature, we highlight a strong positive correlation between viral respiratory infection incidence and ambient PM_2.5_ concentrations and the increase in susceptibility to COVID-19 mortality caused by long term exposure to PM_2.5_, consistent with evidence for the original SARS-Coronavirus during the 2003 outbreak. In fact, fine PM is already known to affect cardiovascular and respiratory morbidity and mortality.

However, we are aware that the phenomenon and the cause and effect relationships are very complex and that our work can only address part of the problem. The cross-sectional nature of the dataset and the use of geographically aggregated information in the epidemiological model does not allow concluding a causal effect exists. In our opinion, the robust evidence in the paper shows that the relationship between PM2.5 and COVID-19 related excess deaths goes far beyond a simple geographical correlation, and further research is needed to explore the causal effect more in depth, when reliable time series data are available.

In fact, our paper does not deal with the spread of contagion and the dynamics linked to it, also because, as we underlined, such analysis would require time-series data, a different econometric methodology, and the identification of the exogenous Coronavirus insurgence in Northern Italy. To the latter purpose, the spread of the pandemic incorporates two different dynamics: (1) on the one hand, the dynamics of the spread of the contagion requires further information to be investigated such as its genesis, the type of virus, and setting of the first outbreak; (2) the effects of the lockdown changed (or partially blocked) the contagion in an asymmetric way. In addition to this, of course, there are other elements that should be investigated, such as additional variables about health data, mobility, and so forth.

Our results reinforce the need to adopt environmental policies that would not only reduce the impact of pollution on the health of citizens and workers but would contribute to smooth the negative effects of a (future) pandemics, avoiding collapses of health systems. Indeed, recent studies show that in addition to chronic lung inflammation, environmental air pollutant concentrations can exacerbate the effects of increasingly frequent one-shot systemic shocks, which in turn are also caused by environmental factors. In this regard, sustainable and decarbonization policies such as the Green New Deal, conceived as long-term policies, should be accelerated.

## Appendix 1

See Table 5.

## Appendix 2

See Table 6.

## Appendix 3

See Table 7.

## Appendix 4

Data comparison from different official sources.

Table 8 reports the excess deaths data as reported by the Italian Bureau of Statistics (ISTAT) along with the official covid19 statistics as indicated by Protezione Civile Italiana (PC). ED-I stands for Excess deaths reported by ISTAT, calculated as the sum of deaths (from all causes) between January 1 and March 31 (column 1) or April 30. 2020 (column 3) with respect to the average value in 2015–2019 (same months). The difference between column 1 and column 2 involves the sampling base. In fact, as long as ISTAT was upgrading the deaths data, it was both enlarging the sample of municipalities and correcting the past figures; see column 4 to 6: TD-I (a) stands for Total deaths reported by ISTAT as the sum of total deaths from January 1 to March 31, 2020 in the initial sample (column 4), TD-I (b) for the updated sample (column 5) and TD-I (c) for the latter release. It turned out that the number of ISTAT excess deaths increased by 97% with this revision (compare column 1 and column 2). It further increased by 52% on April 30 (compare column 2 and column 3) due to corrections, enlargement and new cases.

**Table 8:** Regional comparison of death data from different sources

Regions	(1)	(2)	(3)	(4)	(5)	(6)	(7)	(8)	(9)	(10)	(11)
	ED-I (a) 1 J-31 M	ED-I (b) 1 J-31 M	ED-I (c) 1 J-30A	TD-I (a) 1 J-31 M	TD-I (b) 1 J-31 M	TD-I (c) 1 J-30A	D-PC 31 M	D-PC 30A	(1)/(7)	(2)/(7)	(3)/(8)
Emilia-Romagna	1874	3251.5	4525	8433	16,879	22,142	1644	3551	1.14	1.98	1.27
Friuli-Venezia Giulia	44	55	255	353	1596	5332	113	289	0.39	0.49	0.88
Liguria	65	837.75	1454	3982	6187	9193	428	1167	0.15	1.96	1.25
Lombardy	8539	15,771.75	23,329	26,749	40,969	58,882	7199	13,772	1.19	2.19	1.69
Piemonte	816	2216.75	3547	5566	12,637	21,931	854	3066	0.96	2.60	1.16
Trentino-Alto Adige	132	416.25	1048	551	1737	4286	240	693	0.55	1.73	1.51
Valle d’Aosta	25	117	128	184	541	622	56	137	0.45	2.09	0.93
Veneto	529	1002	1723	4884	12,645	18,248	477	1459	1.11	2.10	1.18
total	12,024	23,668	36,009	50,702	93,191	140,636	11,011	24,134	1.09	2.15	1.49
municipalities	n.a.	6866	7270	n.a.	6866	7270	–	–	–	–	–

*ED-I (a)* excess deaths reported by ISTAT January 1-March 31 2020 with initial sample; *ED-I (b)* excess deaths by ISTAT January 1-March 31 with enlarged sample; *ED-I (c)* excess deaths by ISTAT, latest release; *TD-I (a)* total deaths by ISTAT January 1-March 31 with initial sample; *D-PC* number of deaths with or from Covid-19 registered by Protezione Civile Italiana (PC). Total municipalities in Northern Italy: 7904

D-PC stands for number of deaths with or from Covid-19 reported by the national department Protezione Civile Italiana (Italian Civil Protection, which is a department of the Presidency of the Italian Council of Ministers) over January 1-March 31, 2020 (column 7) and January 1-April 30, 2020 in column 8. The official D-PC data are available only at the regional level and they are officially released by the health departments of the Regional administrations. In the period March 31 and April 30, PC registered a 119% increase in Covid-19 deaths.

ISTAT and PC department therefore collect data independently. The key difference between ED-I and D-PC lies in how PC recognizes fatalities as Covid-19 related: only patients known to have tested positive to SARS-COV-2 got registered under this nomenclature. On the other hand, ED-I is just a mathematical construct that takes into account all deaths in a given municipality, regardless the cause. Due to difficulties in providing timely screenings and accurate testing during the peak of the pandemics, it is likely that official figures from PC might have been underestimated between early January and mid-April. This is particularly true for the most affected areas. Consequently, the discrepancies between ED-I and D-PC can be either moderate or strong, as we observe for Lombardy (where ED-I is roughly 69% higher than D-PC) and Trentino Alto-Adige. Last, the unlikely event of ED-I lying below D-PC is due the fact that ISTAT initially collected figures for only a subsample of municipalities (i.e. those recording a percentage of excess deaths in 2020 greater than 20%) leaving out quite a lot of statistical units (for example, Friuli-Venezia Giulia, which had initially the biggest discrepancy (ratio 0,39) reported data for a very small number of municipalities to ISTAT). However, with some delay, ISTAT has been upgrading the sample, such that in June it covered about all municipalities.

In our paper, we use the updated data ED-I 1 J-30A (column 3), disaggregated at the municipality level. Column (9) shows that the ratio of total ED-I and D-PC is about 1.09, with mean ratio equal to 0.74 and standard deviation equal to 0.4. Underestimation of cases in the initial sampling base concerned all Northern Italian regions except Emilia Romagna, Lombardy and Veneto (the biggest regions in terms of population and Covid-19 cases).

The ratio of total ED-I (b) and D-PC (b) in column (10) is about 2.1, with a mean ratio equal to 1.89 and standard deviation 0.6. One month later, the total ratio decreases to 1.5—column (11)—with mean ratio 1.24 and standard deviation 0.27. In the latter case, a slight underestimation of the registered cases concerns only FVG and Valle d’Aosta.

In any case, since Lombardy has the biggest discrepancy on April 30 (ratio 1,69) we also run our regressions excluding Lombardy (i.e. taking all Lombardy municipalities off) and the results are robust, reporting a significant higher-than-1 relative risk ratio for the exposure variable. The latter results are available upon request.

## Footnotes

1 Data from Johns Hopkins coronavirus resource center, updated July 10.

2 At January 1st 2019, source: Italian Bureau of Statistics—ISTAT.

3 Different components of gas emissions (e.g. nitrogen dioxide, carbon monoxide), derive from many activities like traffic congestion, house heating, agricultural and husbandry practices, as well as industrial combustion. Concentrations are characterized by seasonality, with high levels in Winter, and weather conditions, however the 2020 lockdown measures reduced substantially those derived from traffic but not those derived from agricultural activities (ARPA Lombardia 2020).

4 Data from the annual report on air quality in Italian cities by Legambiente, available at https://www.legambiente.it/wp-content/uploads/Malaria2019_dossier.pdf.

5 https://ec.europa.eu/eurostat/web/nuts/background.

6 We also replicate the analysis using other trend-stationary models (i.e. universal kriging) and different covariance functions; these extensions are discussed in the robustness check section.

7 We omit average humidity because the variable shows a too strong linear correlation (ρ= 0.98) with temperature in our sample observations, and its inclusion would cause severe imperfect collinearity in the model.

8 The test is performed using queen-contiguity based spatial weights.

9 The remaining regions in the base category are Liguria, Valle d’Aosta, Trentino Alto Adige e Friuli-Venezia Giulia. We performed the analysis also including dummy variables for the remaining regions and the results do not change (the related coefficients are jointly insignificant). Results are available upon request.

10 IRRs indicate the % change in Covid-related mortality for each one-unit increase in PM2.5 concentration.

11 van Donkelaar, A., R. V. Martin, M. Brauer, N. C. Hsu, R. A. Kahn, R. C. Levy, A. Lyapustin, A. M. Sayer, and D. M. Winker. 2018. Global Annual PM2.5 Grids from MODIS, MISR and SeaWiFS Aerosol Optical Depth (AOD) with GWR, 1998–2016. Palisades, NY: NASA Socioeconomic Data and Applications Center (SEDAC). 10.7927/H4ZK5DQS. Accessed 01 Mar 2020. See van Donkelaar et al. (2016) for further reference.

12 2016 Annual average concentration in μg/m^3^ of Pm 2.5 processed from the Data Integration Model for Air Quality (DIMAQ) (Shaddick et al. 2018) from the WHO website.

13 Monitoring Air quality data for PM2.5 annual average concentration for 2016 and 2017, interpolated in a ‘regression-interpolation-merging mapping’ (Horálek et al. 2018).
